# Associations between sugar-sweetened beverages consumption, duration of physical exercise, and depressive symptoms among Tibetan university students at high altitude

**DOI:** 10.3389/fpsyg.2024.1439451

**Published:** 2024-08-20

**Authors:** Yang Yang, Jia Liu, Duo Dai

**Affiliations:** ^1^Physical Education Institute of Huanghuai University, Zhumadian, China; ^2^Physical Education Institute of Sichuan Minzu College, Kangding, China

**Keywords:** sugar-sweetened beverages, duration of physical exercise, depressive symptoms, Tibetan, high altitude

## Abstract

**Background:**

Depressive symptoms have become a public health issue of common concern in countries all over the world, and have many negative impacts on university students’ study and life. Depressive symptoms are influenced by many factors, including sugar-sweetened beverages (SSBs) consumption and duration of physical activity. However, no study has been conducted on the association between sugar-sweetened beverage consumption, duration of physical exercise, and depressive symptoms among Tibetan university students at high altitudes.

**Methods:**

In this study, a self-assessment survey of SSBs consumption, duration of physical exercise, and depressive symptoms was conducted on 6,259 (2,745 boys, 43.86%) Tibetan university students aged 19–22 years in Lhasa and Ganzi areas, China, using stratified whole population sampling. The associations were also analyzed using one-way analysis of variance, binary logistic regression analysis, and ordered logistic regression analysis in the generalised linear model.

**Results:**

The proportions of mild depression symptoms, moderate depression symptoms, and major depressive symptoms among Tibetan boys university students in high-altitude areas of China were 18.0, 22.9, and 1.5%, respectively; the proportions of girls students were 20.1, 21.9, and 1.5%, respectively, 1.5%, and the differences in the detection rates of depressive symptoms between sex were statistically significant (*χ*^2^ value = 14.253, *p* < 0.01). Ordered logistic regression analyses showed that using duration of physical exercise >60 min/d and SSBs ≤1 times/week as the reference group, the duration of physical exercise <30 min/d and SSBs ≥5 times/week groups had the highest risk of developing depressive symptoms was the highest risk (OR = 6.98, 95% CI: 5.05–9.65; *p* < 0.001).

**Conclusion:**

This study confirmed that there was a positive association between SSBs consumption and depressive symptoms and a negative association between the duration of physical exercise and depressive symptoms among Tibetan university students at high altitudes in China. In the future, SSBs consumption should be effectively controlled and the duration of physical exercise should be increased to reduce the occurrence of depressive symptoms and promote the physical and mental health of Tibetan university students in high-altitude areas.

## 1 Introduction

With the rapid development of social economy, lifestyle, and information technology, depressive symptoms among university students show a high prevalence, and have become an important mental public health problem. According to the survey, the detection rate of depressive symptoms among Chinese university students is as high as 23.24–39.51%, which is higher than the reporting rate of 8–19% in Europe and North America, and continues to rise and show a trend of aging (Shorey et al., 2022; Ting, 2023). Depression is the number one cause of disability worldwide, affecting more than 350 million people of all ages (Alves et al., 2021). The emergence of depressive symptoms, if not promptly intervened or treated, will develop into depression, which will have serious negative impacts on life and learning (Ibrahim et al., 2013). University students are facing the double pressure of study and employment, and the prevalence of depressive symptoms is constantly increasing (Pacheco et al., 2017; Shao et al., 2020). Several previous studies have confirmed that the factors affecting the occurrence of depressive symptoms in university students are multifaceted, including family environment, lifestyle, dietary behaviors, screen time, exercise habits, ethnicity, altitude, and other factors, and that all of the above factors will have an impact on the depressive symptoms of university students (Kaya et al., 2021; Lugata et al., 2021; Gupta et al., 2022; Ortiz-Prado et al., 2022). It has also been shown that dietary behaviors and exercise habits, as important factors affecting the health of university students, are also important factors affecting the mental health of university students, especially in the occurrence of depressive symptoms (Ramon-Arbues et al., 2019). It is therefore important to focus on the effects of eating behavior and duration of physical exercise on depressive symptoms.

Worldwide sugar-sweetened beverage consumption has been increasing over the past few decades, with an increase of more than 40% from 1990 to 2016 (Egnell et al., 2017). China is no exception, with the proportion of SSBs consumed by university students rising from 72.6 to 90.3% between 2004 and 2011 (Guo et al., 2021). The increase in SSBs consumption poses a serious threat to the physical and mental health development of university students. For example, it causes weight gain, increased prevalence of type 2 diabetes, and increased risk of hypertension (von Philipsborn et al., 2019; Grossman et al., 2022). University students, as one of the major consumer groups of SSBs, have seen a sustained increase in their SSBs consumption, which has important negative impacts on their physical and mental health. There is growing evidence that SSBs consumption negatively affects the mental health of university students. A meta-analysis also showed an association between increased SSBs consumption and increased prevalence of depressive symptoms (Hu et al., 2019). A survey of adults in the United States also showed that the prevalence of mental health problems was 26% higher among adults with increased consumption of SSBs compared with those who consumed SSBs once a day (Freije et al., 2021). In addition, the only study that has also shown an association between excessive consumption of SSBs and increased psychiatric morbidity has also been found in the Chinese adult population (Yu et al., 2015). Other studies have confirmed that the mental health of university students is strongly linked to their exercise habits, in addition to a strong association with SSBs.

Past studies have shown that there is a strong relationship between the duration of physical exercise and the development of physical and mental health among university students and that maintaining a certain duration of physical exercise is important for promoting physical and mental health (Grasdalsmoen et al., 2020). Research has shown that maintaining a regular duration of physical exercise is important for promoting the psychological health of university students and that physical exercise can alleviate many of the psychological pressures brought about by academics (Sampasa-Kanyinga et al., 2020). It has also been shown that physical exercise can promote the secretion of dopamine in the body, prompting exercisers to achieve the effects of physical and mental pleasure and promote the development of mental health (Noetel et al., 2024). In addition, a meta-analysis also showed that physical exercise has a positive effect on the mental health of adults, especially on the alleviation of depressive symptoms, and it is recommended that adults should maintain a reasonable duration of physical exercise to better promote the development of mental health (Recchia et al., 2022). However, previous studies on the duration of physical exercise and mental health have mainly focused on university students in plains areas, while fewer investigations and studies have been conducted on university students in high-altitude areas (Grasdalsmoen et al., 2020). In addition, even fewer studies have been conducted on groups of hereditary Tibetan college students at high altitudes.

China’s Qinghai-Tibetan Plateau region is a typical high-altitude region in the world, with an average altitude of more than 3,000 meters above sea level, and its high altitude leads to a special environment that has an important impact on the physical and mental health of university students in this region (Zhao et al., 2023). High-altitude areas have different lifestyles, exercise behaviors, and dietary behaviors from those in the plains, due to the scarcity of oxygen and long hours of sunshine. The Tibetans, as one of the world’s typical ethnic groups living at high altitude, have a long history of living at high altitude and have lived at high altitude for a long time, with typical plateau characteristics (Wang et al., 2023). High altitude and hypoxic environments are prone to neurological symptoms such as headache and dizziness, it also causes changes in the immune system, which can lead to various types of mental disorders (Falla et al., 2021; You et al., 2024). Past studies have shown that there are fewer studies on the effects of high altitude and hypoxic environments on psychiatric symptoms (Hufner et al., 2019). In addition, there are fewer previous studies on depressive symptoms among Tibetan university students at high altitudes in China. To the best of our knowledge, no studies have been found on the association between SSBs consumption, duration of physical exercise, and depressive symptoms in Tibetan university students at high altitudes. However, it is unclear whether depressive symptoms among Tibetan university students at high altitude differ from those on the plains, and whether they are also influenced by SSBs consumption and duration of physical activity. In this study, we conducted a cross-sectional survey on SSBs consumption, duration of physical exercise, and depressive symptoms among 6,259 Tibetan university students at high altitude in China, and analyzed the associations among them, to provide references for the development of physical and mental health and interventions for Tibetan university students at high altitude.

## 2 Methods

### 2.1 Participants

In this study, participants were sampled using stratified random whole cluster sampling with the class as the smallest unit of sampling. The sampling of participants for this study was divided into three stages. In the first stage, Lhasa (3,650 m) in Tibet and Ganzi (3,410 m) in Sichuan, China were used as the sampling regions for the participants of this study. In the second stage, one university was randomly selected as a test school in each region. In the third stage, 15 teaching classes were randomly selected in each grade from freshman to senior year in each school, for a total of 40 teaching classes.

Eligible Tibetan university students in the class served as participants in this study. The inclusion criteria for this study were: living in the region for at least 3 years and above, having both parents of Tibetan ethnicity, and volunteering to be surveyed for this study. The exclusion criteria for this study were: less than 3 years of residence at high altitude, one or both parents were non-Tibetan, did not sign an informed consent form, and were unwilling to be evaluated for this study. Eventually, a total of 6,512 Tibetan university students aged 19–22 years old in 120 teaching classes were tested in this study. After the test, 253 invalid data were excluded, and finally, 6,259 valid data were returned (2,745 boys students, 43.86%), with a valid questionnaire return rate of 96.11%.

**Figure 1:**
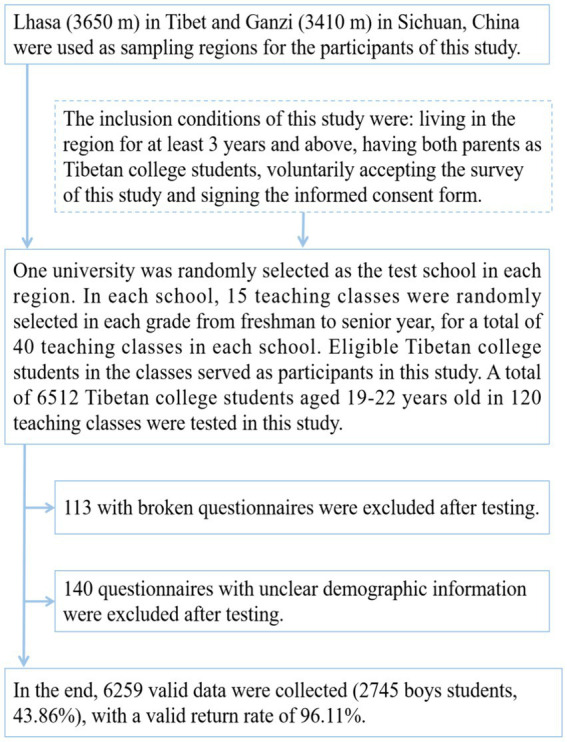
Flowchart of sampling participants from Tibetan university students in high-altitude areas of China.

The questionnaires were administered by trained and qualified teachers. The questionnaires were administered on-site by entering each classroom separately. Participants were asked to sign an informed consent form before the questionnaire was administered. The questionnaires were distributed, filled out on the spot, and returned on the spot. Each questionnaire took about 10–15 min to complete. When the questionnaires were retrieved, participants were checked for completeness of the questionnaires to ensure that the questionnaires were filled out in a standardized and clear manner. The specific sampling process is shown in Figure 1.

This study was conducted by the Declaration of Helsinki, and approved by the Human Ethics Committee of Huanghuai University (2023457412).

### 2.2 Depressive symptoms

In this study, depressive symptoms of Tibetan university students at high altitude were assessed using the Self-rating Depression Scale (SDS; Zung, 1971). The SDS is a tool for measuring depression. Developed in 1965 by Professor William W. K. Zung of Duke University, USA. It includes 20 items, each item consisting of a 4-point scale. It includes 2 items for psychotic-affective symptoms, 8 items for somatic disorders, 2 items for psychomotor disorders, and 8 items for depressive mental disorders. The scale is easy to use and visually reflects participants’ subjective feelings. The scale has been widely used in the Chinese adult population and has good reliability, with Cronbach’s alpha coefficients of 0.86–0.91 (Dunstan et al., 2017; Yan et al., 2024). The total score of the 20 entries was 80, and the standard score was obtained by multiplying 80 points by 1.25. Based on the results of the Chinese norm, the cut-off value of the SDS standard score was 53 points, <53 points was no depressive symptoms, among which 53–62 points were mild depressive symptoms, 63–72 points were moderate depressive symptoms and 73 points or more was major depressive symptoms. Symptoms (Quanquan and Shengli, 2012). In this study, participants with SDS scores ≥53 were defined as having depressive symptoms.

### 2.3 Sugar-sweetened beverages

Self-assessment of SSBs consumption among Tibetan university students at high altitudes in this study was conducted using the Beverage Intake Questionnaire (BEVQ15) questionnaire (Hedrick et al., 2012). The questionnaire was designed to assess participants’ consumption of 15 SSBs in the past 30 days. The scale has been widely used in the Chinese university student population and has good reliability and validity (Zhuang et al., 2021; Shi et al., 2023). The frequency of SSBs consumption and the number of milliliters consumed per occasion were included. The types of SSBs included fruit juice drinks, chocolate drinks, milk-based drinks, flavored fruit juices, black coffee-based drinks, nut-based drinks, carbonated drinks, functional drinks, and flavored milk tea drinks. Based on participants’ SSBs consumption within the past 30 days, intake of SSBs was categorized into 7 categories: never or <1 time/week, 1 time/week, 2–3 times/week, 4–6 times/week, 1 time/day, 2 times/day, and >3 times/day. Each intake of SSBs was recorded in milliliters (mL) for each reported beverage. The frequency of SSBs consumption was categorised in this study as ≤1 times/week, 2–4 times/week, and ≥5 times/week. The number of millilitres per SSBs consumption was calculated based on a can of Coke 330 ml (Shi et al., 2023).

### 2.4 Duration of physical exercise

The duration of physical exercise in this study was assessed using the China National Survey on Students’ Constitution and Health (CNSSCH) questionnaire (CNSSCH Association, 2022). The questionnaire assesses the duration of physical exercise every 5 years for students aged 7–22 years nationwide in China. The investigation of the duration of physical exercise in this study focuses on the daily duration of moderate- to high-intensity physical exercise, such as ball games, exercise in spaced-net sports, hiking, brisk walking, running, cycling, skating, skiing, and other sports. Sweating is achieved during exercise. The present study focused on the duration and frequency of the participant’s participation in various types of physical activities during the past 7 days, including the weekday hours from Monday to Friday and 2 days on Saturday and weekend. From this, the average duration of physical activity per day was calculated for the participants over the past 7 days. By referring to the classification criteria of international studies and combining them with the reality of the study, the duration of physical activity of the participants was classified into three groups: <30 min/d, 30–60 min/d, and > 60 min/d (Zhang et al., 2020).

### 2.5 Covariates

The covariates investigated in this study included age, an only child, socioeconomic status (SES), sleep quality, screen time, and body mass index (BMI). Participants ages and their only children were obtained by direct assessment of the questionnaire was calculated using indicators such as parental literacy, parental occupation, and family income to measure participants’ family socioeconomic status. In this study, the SES scores were calculated according to the method of calculating SES in the Programme for International Student Assessment (PISA), and the SES scores in this study were classified into three classes according to their percentile rank: lower (<20th), middle (20–80th), and high (>80th; Turner and Adams, 2007). Sleep quality was assessed with the Pittsburgh sleep quality index (PSQI; Zitser et al., 2022). The PSQI is a widely used self-assessment scale for assessing sleep quality, which consists of 19 self-rated questions and 5 questions rated by sleep peers, but only 19 self-rated questions are used in the actual scoring process. These 19 questions are distributed over 7 different factors: sleep quality, time to sleep, sleep duration, sleep efficiency, sleep disorders, hypnotic drug use, and daytime dysfunction. Each factor has a specific scoring method, and the scores of each factor are eventually accumulated to obtain a total PSQI score, which ranges from 0 to 21, with higher scores indicating poorer sleep quality. In this study, PSQI ≤5 points was defined as “good” sleep quality and PSQI >5 points was defined as “poor” sleep quality. Screen time was measured as the average time spent on mobile phones, tablet computers, and TVs in the past 7 days, including screen time from Monday to Friday, Saturday, and Sunday. The average daily screen time over the past 7 days was calculated sequentially. By the relevant international standards and the relevant research literature, the screen time of the participants in this study was classified into ≤2 h/d and >2 h/d (Tambalis et al., 2020). BMI was calculated based on height and weight assessments. Participants height and weight were tested according to the testing methods and instruments required by the China National Survey on Students’ Constitution and Health (CNSSCH; CNSSCH Association, 2022). Height was assessed to the nearest 0.1 cm and weight to the nearest 0.1 kg. The equation for calculating BMI is weight (kg)/height (m)^2^. Based on the BMI classification standard for Chinese adults. In this study, BMI <18.5 kg/m^2^ was assessed as “slimmer,” 18.5–23.9 kg/m^2^ was assessed as “normal” and 24.0–27.9 kg/m^2^ is assessed as “overweight,” and ≥28.0 kg/m^2^ is rated as “obese” (Wang et al., 2021).

### 2.6 Statistical analysis

The distribution of different categories of Tibetan university students at high altitudes was expressed as a percentage (%). A comparison of different SSBs and duration of physical exercise university students with depressive symptoms was performed using the chi-square test (Chen et al., 2022). The associations of SSBs and duration of physical exercise among Tibetan university students at high altitudes with depressive symptoms were analyzed using stratified logistic regression analysis (Chen et al., 2022). After stratification according to sex, the presence of depressive symptoms among Tibetan university students was used as the dependent variable, and SSBs and duration of physical exercise were used as independent variables for the analysis. The crude model was not adjusted for any covariates, Model 1 was adjusted for age, an only child, and SES, and Model 2 was further adjusted for sleep quality, screen time, and BMI based on Model 1. In addition, to further analyze the association between SSBs, duration of physical exercise, and depressive symptoms, analyses were conducted using ordered logistic regression analysis in generalized linear models. The analyses were performed after stratification by sex, with depressive symptoms as the dependent variable and SSBs and duration of physical exercise as the independent variables. Ordered Logistic Regression adjusted for age, an only child, SES, sleep quality, screen time, and BMI. Analyses reported odds ratios (OR) and 95% confidence intervals (CI), respectively. Data were analyzed using SPSS 25.0 (SPSS Inc., Chicago, IL, USA) software. A two-sided test level of *α* = 0.05 was used.

## 3 Results

In this study, a cross-sectional survey of SSBs consumption, duration of physical exercise, and depressive symptoms was conducted among 6,259 Tibetan university students at high altitudes. The mean age of the participants was (20.18 ± 1.03) years. The results of this study showed that the proportions of mild depression symptoms, moderate depression symptoms, and major depressive symptoms among boys Tibetan university students at high altitude were 18.0, 22.9, and 1.5%, respectively; and the proportions among girls students were 20.1, 21.9, 1.5%, and the difference in the detection rate of depressive symptoms between different sex was statistically significant (*χ*^2^ value = 14.253, *p* < 0.01). The basic situation of the distribution of different categories of Tibetan university students at high altitudes is shown in Table 1.

**Table 1:** The basic distribution of different categories of Tibetan university students in high-altitude areas (%).

Classification	Boys	Girls	Total
*N*	2,745	3,514	6,259
**Age**			
19 yrs	785 (28.6)	1,210 (34.4)	1,995 (31.9)
20 yrs	836 (30.5)	1,182 (33.6)	2018 (32.2)
21 yrs	635 (23.1)	758 (21.6)	1,393 (22.3)
22 yrs	489 (17.8)	364 (10.4)	853 (13.6)
**An only child**			
Yes	900 (32.8)	698 (19.9)	1,598 (25.5)
No	1,845 (67.2)	2,816 (80.1)	4,661 (74.5)
SES			
Lower	475 (17.3)	493 (14.0)	968 (15.5)
Middle	1,898 (69.1)	2,534 (72.1)	4,432 (70.8)
High	372 (13.6)	487 (13.9)	859 (13.7)
**Sleep quality**			
Good (≤5 points)	834 (30.4)	880 (25.0)	1714 (27.4)
Poor (>5 points)	1911 (69.6)	2,634 (75.0)	4,545 (72.6)
**Screen time**			
≤2 h/d	777 (28.3)	844 (24.0)	1,621 (25.9)
>2 h/d	1,968 (71.7)	2,670 (76.0)	4,638 (74.1)
**BMI**			
Slimmer	294 (10.7)	738 (21.0)	1,032 (16.5)
Normal	1,416 (51.6)	1,884 (53.6)	3,300 (52.7)
Overweight	525 (19.1)	281 (8.0)	806 (12.9)
Obese	510 (18.6)	611 (17.4)	1,121 (17.9)
**SSBs consumption**			
≤1 times/week	1,541 (56.1)	2,017 (57.4)	3,558 (56.8)
2–4 times/week	562 (20.5)	912 (26.0)	1,474 (23.6)
≥5 times/week	642 (23.4)	585 (16.6)	1,227 (19.6)
**Duration of physical exercise**			
<30 min/d	1,820 (66.3)	2,833 (80.6)	4,653 (74.3)
30–60 min/d	671 (24.4)	489 (13.9)	1,160 (18.5)
>60 min/d	254 (9.3)	192 (5.5)	446 (7.1)
**Depressive symptoms**			
No depressive symptoms	1,581 (57.6)	1,955 (55.6)	3,536 (56.5)
Mild depression symptoms	495 (18.0)	766 (21.8)	1,261 (20.1)
Moderate depression symptoms	629 (22.9)	741 (21.1)	1,370 (21.9)
Major depressive symptoms	40 (1.5)	52 (1.5)	92 (1.5)

*N*, Numbers; yrs, years; SES, Socioeconomic Status; BMI, Body Mass Index; SSBs, Sugar-sweetened beverages; h/d, Hours/day; min/d, Minutes/day.

The results of this study showed that the proportion of major depressive symptoms of Tibetan university students in high-altitude areas of China was 0.9, 1.9, and 2.7% when SSBs≤1 time/week, 2–4 times/week, and ≥5 times/week, respectively. The difference was statistically significant (*χ*^2^ = 23.284, *p* < 0.001). The rates of moderate depression symptoms were 17.1, 22.3, and 35.1%, respectively, and the difference was statistically significant (*χ*^2^ = 172.757, *p* < 0.001). In terms of duration of physical exercise <30 min/d, 30–60 min/d, and >60 min/d, the proportion of major depressive symptoms was 1.7, 0.2, and 2.7%, respectively, and the difference was statistically significant in comparison (*χ*^2^ = 19.442, *p* < 0.001). The percentages of moderate depression symptoms were 23.7, 19.0, and 10.5%, respectively, and the difference was also statistically significant (*χ*^2^ = 48.385, *p* < 0.001). The proportion of mild depression symptoms was 21.5, 17.8, and 12.1%, respectively, and the difference was statistically significant in comparison (*χ*^2^ = 27.427, *p* < 0.001). A comparison of SSBs consumption, duration of physical exercise, and depressive symptoms among Tibetan university students at high altitude by sex is shown in Table 2.

**Table 2:** Univariate analysis of SSBs consumption, duration of physical exercise, and depressive symptoms among Tibetan university students in high-altitude areas.

Classification		N	No depressive symptoms			Mild depression symptoms			Moderate depression symptoms			Major depressive symptoms		
			*N* (%)	*χ* ^2^	*p*- value	*N* (%)	*χ* ^2^	*p*- value	*N* (%)	*χ* ^2^	*p*- value	*N* (%)	*χ* ^2^	*p*- value
**Boys**														
SSBs	≤1 times/week	1,541	983 (63.8)	71.803	<0.001	273 (17.7)	1.23	0.541	277 (18.0)	60.618	<0.001	8 (0.5)	23.534	<0.001
	2–4 times/week	562	314 (55.9)			97 (17.3)			139 (24.7)			12 (2.1)		
	≥5 times/week	642	284 (44.2)			125 (19.5)			213 (33.2)			20 (3.1)		
Duration of physical exercise	<30 min/d	1820	999 (54.9)	23.537	<0.001	332 (18.2)	0.273	0.872	452 (24.8)	20.802	<0.001	37 (2.0)	12.992	0.001
	30–60 min/d	671	404 (60.2)			120 (17.9)			146 (21.8)			1 (0.1)		
	>60 min/d	254	178 (70.1)			43 (16.9)			31 (12.2)			2 (0.8)		
**Girls**														
SSBs	≤1 times/week	2017	1,257 (62.3)	129.11	<0.001	404 (20.0)	8.728	0.013	333 (16.5)	117.442	<0.001	23 (1.1)	4.278	0.118
	2–4 times/week	912	487 (53.4)			219 (24.0)			190 (20.8)			16 (1.8)		
	≥5 times/week	585	211 (36.1)			143 (24.4)			218 (37.3)			13 (2.2)		
Duration of physical exercise	<30 min/d	2,833	1,472 (52)	90.425	<0.001	669 (23.6)	39.653	<0.001	651 (23.0)	35.281	<0.001	41 (1.4)	23.784	<0.001
	30–60 min/d	489	328 (67.1)			86 (17.6)			74 (15.1)			1 (0.2)		
	>60 min/d	192	155 (80.7)			11 (5.7)			16 (8.3)			10 (5.2)		
**Total**														
SSBs	≤1 times/week	3,558	2,240 (63.0)	193.476	<0.001	677 (19.0)	6.49	0.039	610 (17.1)	172.757	<0.001	31 (0.9)	23.284	<0.001
	2–4 times/week	1,474	801 (54.3)			316 (21.4)			329 (22.3)			28 (1.9)		
	≥5 times/week	1,227	495 (40.3)			268 (21.8)			431 (35.1)			33 (2.7)		
Duration of physical exercise	<30 min/d	4,653	2,471 (53.1)	102.262	<0.001	1,001 (21.5)	27.427	<0.001	1,103 (23.7)	48.385	<0.001	78 (1.7)	19.442	<0.001
	30–60 min/d	1,160	732 (63.1)			206 (17.8)			220 (19.0)			2 (0.2)		
	>60 min/d	446	333 (74.7)			54 (12.1)			47 (10.5)			12 (2.7)		

N, Numbers; SSBs, Sugar-sweetened beverages.

Logistic regression analyses were conducted with the presence of depressive symptoms among Tibetan university students in high-altitude areas of China as the dependent variable, and SSBs consumption and duration of physical exercise as the independent variables, stratified by sex. The crude model was not adjusted for any covariates, Model 1 was adjusted for age, an only child, and SES, and Model 2 was further adjusted for sleep quality, screen time, and BMI on the basis of Model 1. Overall, the results showed that, compared with Tibetan university students at high altitude with SSBs ≤1 time/week as the reference group, the ratio of the occurrence of depressive symptoms was higher (*p* < 0.001) for university students with SSBs of 2–4 times/week (OR = 1.32, 95% CI: 1.15–1.52), and the ratio of the occurrence of depressive symptoms was higher (*p* < 0.001) for university students with SSBs ≥5 times/week (OR = 2.06, 95%CI: 1.76 ~ 2.40) had a higher ratio of developing depressive symptoms (*p* < 0.001), and SSBs ≥5 times/week (OR = 2.06, 95%CI: 1.76–2.40) had a higher ratio of developing depressive symptoms (*p* < 0.001). Compared with Tibetan university students at high altitude whose duration of physical exercise was >60 min/d as the reference group, the ratio of depressive symptoms was higher among those whose duration of physical exercise was 30–60 min/d (OR = 1.40, 95%CI: 1.05–1.87), and the ratio of those whose duration of physical exercise was <30 min/d (OR = 1.91, 95%CI: 1.47–2.48) was higher (*p* < 0.05). The ratios of depression symptoms were higher in university students with a duration of physical exercise of 30–60 min/d (OR = 1.40, 95%CI: 1.05–1.87; *p* < 0.05), and those with a duration of physical exercise of <30 min/d (OR = 1.91, 95%CI: 1.47–2.48; *p* < 0.001). The results of logistic regression analyses of Tibetan university students at high altitudes by sex are shown in Table 3.

**Table 3:** Logistic regression analysis of SSBs consumption, duration of physical exercise, and depressive symptoms of Tibetan university students in high altitude area.

Sex	Classification		Crude model		Model 1		Model 2	
			OR (95% CI)	*p*-value	OR (95% CI)	*p*-value	OR (95% CI)	*p*-value
Boys	SSBs	≤1 times/week	1.00		1.00		1.00	
		2–4 times/week	1.39 (1.14 ~ 1.69)	0.001	1.34 (1.10 ~ 1.63)	0.004	1.17 (0.93 ~ 1.49)	0.183
		≥5 times/week	2.23 (1.85 ~ 2.69)	<0.001	2.22 (1.84 ~ 2.69)	<0.001	1.57 (1.26 ~ 1.96)	<0.001
	Duration of physical exercise	>60 min/d	1.00		1.00		1.00	
		30–60 min/d	1.61 (1.18 ~ 2.20)	0.003	1.72 (1.26 ~ 2.37)	0.001	1.62 (1.12 ~ 2.35)	0.01
		<30 min/d	2.00 (1.50 ~ 2.66)	<0.001	2.06 (1.54 ~ 2.75)	<0.001	1.91 (1.36 ~ 2.68)	<0.001
Girls	SSBs	≤1 times/week	1.00		1.00		1.00	
		2–4 times/week	1.46 (1.25 ~ 1.71)	<0.001	1.45 (1.24 ~ 1.70)	<0.001	1.42 (1.19 ~ 1.69)	<0.001
		≥5 times/week	2.97 (2.45 ~ 3.60)	<0.001	2.99 (2.46 ~ 3.63)	<0.001	2.59 (2.09 ~ 3.22)	<0.001
	Duration of physical exercise	>60 min/d	1.00		1.00		1.00	
		30–60 min/d	2.55 (1.66 ~ 3.91)	<0.001	2.46 (1.60 ~ 3.79)	<0.001	1.29 (0.79 ~ 2.09)	0.306
		<30 min/d	4.80 (3.25 ~ 7.10)	<0.001	4.56 (3.08 ~ 6.77)	<0.001	2.26 (1.45 ~ 3.53)	<0.001
Total	SSBs	≤1 times/week	1.00		1.00		1.00	
		2–4 times/week	1.44 (1.27 ~ 1.62)	<0.001	1.41 (1.24 ~ 1.60)	<0.001	1.32 (1.15 ~ 1.52)	<0.001
		≥5 times/week	2.54 (2.22 ~ 2.90)	<0.001	2.55 (2.23 ~ 2.92)	<0.001	2.06 (1.76 ~ 2.40)	<0.001
	Duration of physical exercise	>60 min/d	1.00		1.00		1.00	
		30–60 min/d	1.90 (1.48 ~ 2.44)	<0.001	1.94 (1.51 ~ 2.50)	<0.001	1.40 (1.05 ~ 1.87)	0.022
		<30 min/d	2.87 (2.29 ~ 3.6)	<0.001	2.83 (2.25 ~ 3.55)	<0.001	1.91 (1.47 ~ 2.48)	<0.001

The crude model was not adjusted for any covariates, Model 1 was adjusted for age, an only child, and SES, and Model 2 was further adjusted for sleep quality, screen time, and BMI based on Model 1. SSBs, Sugar-sweetened beverages.

**Figure 2:**
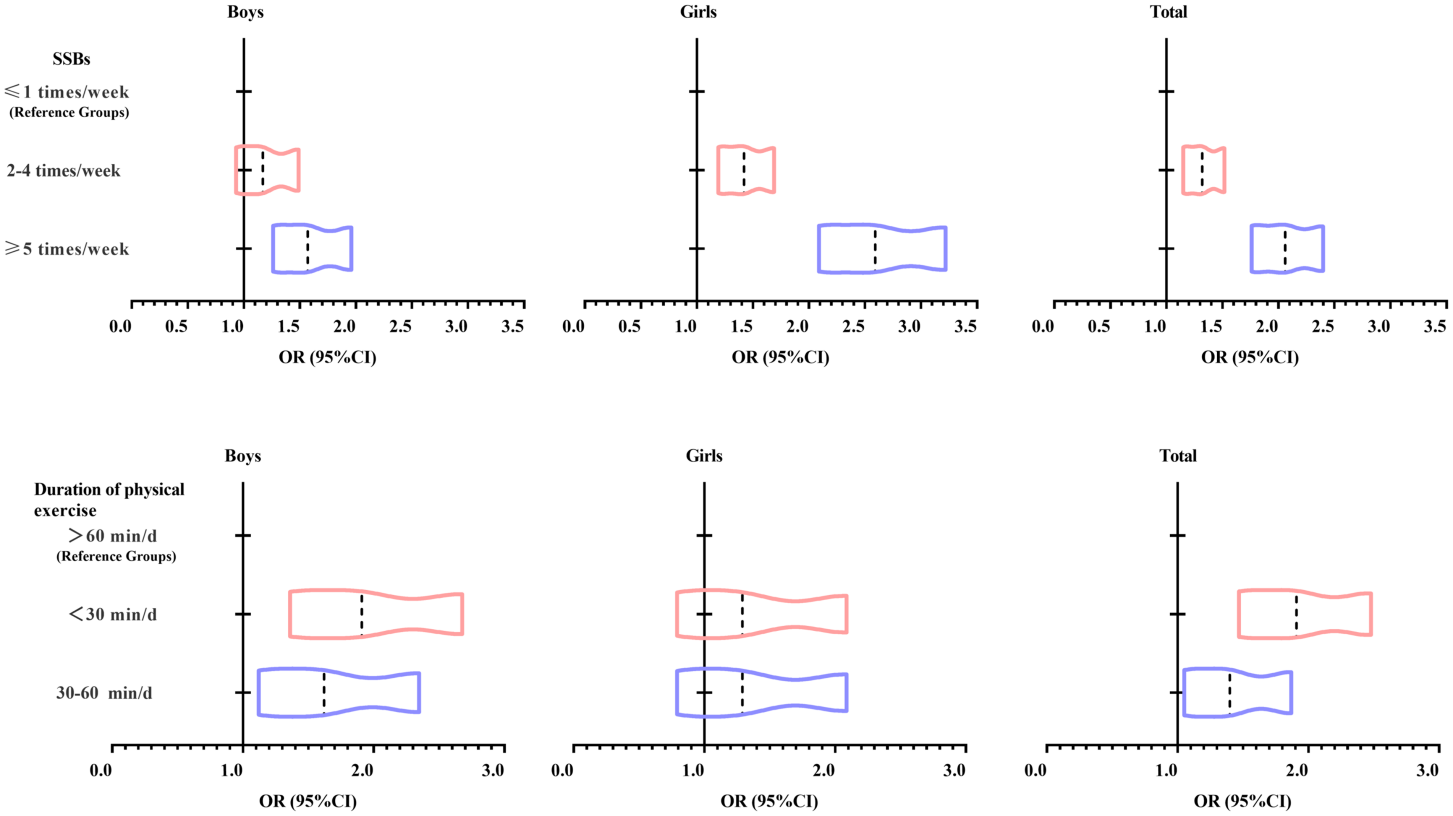
Trends in ORs of logistic regression analyses of SSBs consumption, duration of physical exercise, and depressive symptoms among Tibetan university students at high altitudes. SSBs, Sugar-sweetened beverages; OR (95%CI), Oddratio (95% Confidence Interval).

Figure 2 shows the trend of OR values of logistic regression analysis of SSBs consumption, duration of physical exercise, and depressive symptoms among Tibetan university students at high altitudes. Overall, it can be seen that with the increase in SSBs consumption, the OR value of the occurrence of depressive symptoms of Tibetan university students in high-altitude areas increased. In addition, with the decrease in the duration of physical exercise, the OR value of the occurrence of depressive symptoms among Tibetan university students at high altitudes also increased.

Using the presence of depressive symptoms among Tibetan university students in high-altitude areas of China as the dependent variable, and SSBs consumption and duration of physical exercise as the independent variables, we used ordered logistic regression analyses in a generalized linear model to analyze the relationship between SSBs consumption, after stratifying by sex, duration of physical exercise and depressive symptoms. Ordered Logistic Regression adjusted for age, an only child, SES, sleep quality, screen time, and BMI. Overall, the results showed that compared with the duration of physical exercise >60 min/d and SSBs ≤1 times/week group of university students as the reference group, the duration of physical exercise <30 min/d and SSBs ≥5 times/week group (OR = 6.98, 95% CI: 5.05–9.65) had the highest risk of developing depressive symptoms (*p* < 0.001). In terms of sex, the same trend existed for boys and girls Tibetan university students at high altitudes, and the duration of physical exercise <30 min/d and SSBs ≥5 times/week groups had the highest risk of developing depressive symptoms (*p* < 0.001). Table 4 shows the results of the specific analyses.

**Table 4:** Generalised linear model analysis of SSBs consumption, duration of physical exercise, and depressive symptoms among Tibetan university students in high altitude areas.

Sex	Classification		Ordered logistic regression	
	Duration of physical exercise	SSBs	OR (95% CI)	*p*-value
Boys	>60 min/d	≤1 times/week	1.00	
		2–4 times/week	0.96 (0.45 ~ 2.07)	0.918
		≥5 times/week	2.33 (1.19 ~ 4.56)	0.014
	30–60 min/d	≤1 times/week	1.27 (0.84 ~ 1.91)	0.260
		2–4 times/week	2.46 (1.52 ~ 3.99)	<0.001
		≥5 times/week	4.74 (2.87 ~ 7.83)	<0.001
	<30 min/d	≤1 times/week	1.94 (1.33 ~ 2.82)	0.001
		2–4 times/week	2.39 (1.59 ~ 3.59)	<0.001
		≥5 times/week	3.54 (2.38 ~ 5.26)	<0.001
Girls	>60 min/d	≤1 times/week	1.00	
		2–4 times/week	4.64 (1.98 ~ 10.87)	<0.001
		≥5 times/week	7.15 (1.75 ~ 29.23)	0.006
	30–60 min/d	≤1 times/week	3.87 (1.98 ~ 7.54)	<0.001
		2–4 times/week	6.92 (3.39 ~ 14.13)	<0.001
		≥5 times/week	11.44 (5.17 ~ 25.30)	<0.001
	<30 min/d	≤1 times/week	7.71 (4.12 ~ 14.41)	<0.001
		2–4 times/week	10.33 (5.47 ~ 19.48)	<0.001
		≥5 times/week	20.72 (10.88 ~ 39.46)	<0.001
Total	>60 min/d	≤1 times/week	1.00	
		2–4 times/week	1.74 (1.02 ~ 2.97)	0.042
		≥5 times/week	3.52 (1.93 ~ 6.43)	<0.001
	30–60 min/d	≤1 times/week	1.82 (1.30 ~ 2.56)	0.001
		2–4 times/week	3.38 (2.30 ~ 4.97)	<0.001
		≥5 times/week	6.42 (4.24 ~ 9.72)	<0.001
	<30 min/d	≤1 times/week	3.16 (2.32 ~ 4.30)	<0.001
		2–4 times/week	4.13 (3.00 ~ 5.69)	<0.001
		≥5 times/week	6.98 (5.05 ~ 9.65)	<0.001

Ordered logistic regression adjusted for age, an only child, SES, sleep quality, screen time, and BMI. SSBs, Sugar-sweetened beverages; min/d Minutes/day.

## 4 Discussion

The Tibetan Plateau region of China is a typical high-altitude region in the world, and there are significant differences between this region and the plains in terms of climate and environment. The average altitude of the Tibetan Plateau region is more than 3,000 meters above sea level, and the high altitude brings about thin oxygen in the region, long sunshine hours, strong ultraviolet rays, and the cold and oxygen-deficient environment that has a certain negative impact on the health of the people in the region. Tibetans have lived on the Tibetan Plateau for more than 4,000 years, and have developed typical plateau characteristics as a result of living on the Tibetan Plateau for a long time. There are also studies that show that high-altitude environments are a significant contributor to mental health problems (Chen et al., 2023). Another study also confirmed a significant deterioration in the mental health of soldiers stationed at extreme high altitude for 3 months, which, although it did not reach the level of clinical morbidity, illustrates the negative impact of exposure to high altitude environments on human mental health (Rajesh et al., 2021). The occurrence of sleep dysfunction due to high altitude hypoxic environments is an important cause of the development of depressive symptoms (Liu et al., 2020). In addition, decreased neuronal function due to high-altitude environmental exposure is an important cause of psychological problems (Yan, 2014). The results of this study showed that the prevalence of depressive symptoms among Tibetan university students in high-altitude areas of China was 43.5%, with the prevalence of depressive symptoms among boys Tibetan university students being 42.4% and that of girls Tibetan university students being 44.4%. This result was higher than that of 35.7% for university students in the plains of China (Wenhai et al., 2022). It is also higher than the findings of Shao et al. on depressive symptoms 30.8% of medical university students in the plains of China (Shao et al., 2020). However, the results were not entirely consistent; another survey of university students in the plains of China showed that 48.24% of the university students had depressive symptoms (Malan Lifang et al., 2020). This shows that there are inconsistent findings across studies. On the one hand, there are some differences in the assessment tools used to evaluate depressive symptoms in different studies. On the other hand, differences in testing time and testing area, ethnic composition, etc. are also important reasons for some differences in the results of different studies. However, the present study generally reveals a high level of depressive symptom detection among Tibetan college students at high altitude in China, which is a cause for concern. Studies have shown that long-term residence in high-altitude hypoxic environments affects the mental health of healthy adults, and the reason for this is mainly related to the inhibitory effects of plateau hypoxia on the cerebral nerves (Gao et al., 2023). It has also been confirmed that there is an association between prolonged high altitude exposure and cognitive impairment because as altitude increases, atmospheric pressure decreases and ambient partial pressure of oxygen decreases, resulting in insufficient oxygen supply to maintain brain bioenergetic functioning in individuals exposed at high altitude, leading to brain function and cognitive impairment (Su et al., 2024).

In terms of sex, the detection rate of depressive symptoms among Tibetan university students at high altitudes was higher among girls than boys students in this study, which is consistent with the findings of several studies (Aloufi et al., 2021; Sprung and Rogers, 2021). Overall, it can be seen that the detection rate of depressive symptoms among Tibetan university students in high-altitude areas in China is high, which should be given sufficient attention and concern. Therefore, effectively analyzing the influencing factors affecting depressive symptoms among Tibetan university students in high-altitude areas in China will play a positive role in the development of the psychological health of university students in high-altitude areas.

SSBs consumption has become an important factor affecting the physical and mental health of university students at present. In this study, 23.6 and 19.6% of Tibetan university students at high altitude consumed SSBs 2–4 times/week and ≥5 times/week, respectively. Another study showed that the proportion of “frequent” consumption of SSBs was 21.5% (Ren et al., 2022). This shows that the proportion of SSBs consumed by Tibetan college students at high altitude is higher. Studies have shown that an increase in SSBs consumption is positively correlated with an increase in the detection rate of mental health disorders (Ra, 2022). It has also been shown that increased SSBs consumption will lead to the development of obesity and negatively affect the development of depressive symptoms in university students (Ra, 2023). The results of this study showed that the detection rate of depressive symptoms among Tibetan university students with SSBs ≤1 time/week was lower than that of those with SSBs ≥5 times/week in high-altitude areas of China, and there was a significant difference. This shows that there is a positive correlation between the increase in SSBs consumption and the increase in the detection rate of depressive symptoms. In addition, the same trend was observed for boys and girls. This shows that the effect of SSBs consumption on depressive symptoms does not change according to sex differences. Another survey of Chinese university students also showed that increased SSBs consumption hurt mental health (Xu et al., 2020). In conclusion, the increase in SSBs consumption has a certain impact on the occurrence of depressive symptoms in Tibetan university students at high altitudes, which should be given attention in the future to reduce SSBs consumption and better promote the development of mental health. However, the factors affecting depressive symptoms are multifaceted, and in addition to SSBs consumption affecting university students’ depressive symptoms, physical exercise is also an important factor affecting university students’ depressive symptoms.

The results of this study also showed that the detection rate of depressive symptoms among Tibetan university students at high altitudes tended to increase as the duration of physical exercise decreased. Moreover, the risk of developing depressive symptoms among Tibetan university students at high altitudes increased with the decrease in the duration of physical exercise. This suggests that there is a negative correlation between the duration of physical exercise and the occurrence of depressive symptoms among Tibetan university students at high altitudes. This study showed that the percentage of Tibetan university students at high altitude who had a duration of physical exercise of more than 60 min/d was only 7.1%. In another study, only 5.2% of the students in the plains were found to be physically active. This result is very surprising, because the percentage of Tibetan students who had a duration of physical exercise of more than 60 min/d was higher in the high altitude than in the plains. We believe that there is a correlation between the academic and employment pressures of students in the plains and Tibetan university students at high altitude (Deng et al., 2024). A survey of university students showed that physical exercise has a positive effect on mental health, and active physical exercise can reduce the prevalence of abnormal psychological problems (Henriksson et al., 2022). Another study has also shown that active physical activity in university students can promote blood circulation and the secretion of dopamine hormones in the body, in addition to stimulating neural development and connectivity, releasing neurotrophic factors, and positively affecting the emotional regulation of the exercisers (Dauwan et al., 2021; Ross et al., 2023).

There are certain advantages and limitations in this study. Advantages: First, the participants of this study are Tibetan university students in high-altitude areas in China, and the selection of its participants has typical high-altitude characteristics, and the study has a certain uniqueness, which provides certain theoretical support for the physical and mental health development of Tibetan university students in high-altitude areas. Secondly, this study investigated 6,259 Tibetan university students in high-altitude areas, which is relatively large and representative of the sample size of university students in high-altitude areas. However, this study also has some limitations. On the one hand, this study was a cross-sectional survey, which only allowed us to understand the associations between SSBs consumption, duration of physical exercise, and depressive symptoms among Tibetan university students at high altitudes, but not the causal associations between them. On the other hand, the limited number of covariates included in the logistic regression analyses of this study inevitably had some impact on the results. In the future, prospective cohort studies should be conducted to analyze the causal association between SSBs, duration of physical exercise, and depressive symptoms. At the same time, more covariates should be included, such as whether or not they smoke or drink alcohol, to better analyse the association between SSBs consumption, duration of physical exercise, and depressive symptoms. In addition, this study used self-assessment to assess the participants’ SSBs consumption, duration of physical exercise, and depressive symptoms related to the survey, which inevitably has some bias between the real situation. Although the questionnaire used in this study has been validated in several studies and has good reliability and validity, it also has some bias. In the future, more objective assessment tools should be used to conduct relevant studies.

## 5 Conclusion

The results of this study confirmed that there was a positive association between SSBs consumption and depressive symptoms and a negative association between the duration of physical exercise and depressive symptoms among Tibetan university students at high altitudes in China. Excessive SSBs consumption and reduced duration of physical exercise resulted in a higher prevalence of depressive symptoms among Tibetan university students at high altitudes, and the same trend was found in both boys and girls. In the future, SSBs consumption should be effectively controlled and the duration of physical exercise should be increased in order to reduce the prevalence of depressive symptoms and promote the physical and mental health of Tibetan university students at high altitudes. This study provides guiding recommendations for mental health interventions for Tibetan college students at high altitude, as well as necessary references for the development of high altitude public health policies by the government and the education sector.

## Data Availability

The raw data supporting the conclusions of this article will be made available by the authors, without undue reservation.
